# Enteral feeding protocol: a quality improvement project on feeding interruptions and clinical outcomes in a tertiary intensive care unit

**DOI:** 10.3389/fmed.2025.1562937

**Published:** 2025-11-17

**Authors:** Boon Hui Ng, Wan Rahiza Wan Mat, Rufinah Teo, Azarinah Izaham, Mohd Khazrul Nizar Abd Kader, Jaafar Md Zain, Siti Nidzwani Mohamad Mahdi, Qurratu Aini Musthafa, Aliza Mohamad Yusof

**Affiliations:** 1 Department of Anaesthesia and Intensive Care, Duchess of Kent Sandakan Hospital, Sabah, Malaysia; 2 Department of Anaesthesiology and Intensive Care, Universiti Kebangsaan Malaysia, Kuala Lumpur, Malaysia; 3 Department of Anaesthesiology and Intensive Care, Hospital Canselor Tuanku Muhriz, Kuala Lumpur, Malaysia

**Keywords:** enteral nutrition, intensive care units, critical care, enteral feeding, nutrition therapy, clinical protocol, treatment interruption, critical care outcomes

## Abstract

**Purpose:**

Evaluation of the effectiveness of the feeding protocol in improving feeding interruption (FI) and clinical outcome in critically ill patients.

**Materials and methods:**

This was a single-center, retrospective, and prospective cohort study design evaluating the nutritional characteristics and adequacy, and the causes and clinical outcomes of FI, pre- and post-feeding protocol implementation. The risk factor for ICU mortality was also identified.

**Results:**

In total, 430 patients were included, 217 in the pre-protocol group and 213 in the post-protocol group. After protocol implementation, energy and protein intake significantly improved, and the total target nutrition was achieved. The post-protocol group was prescribed a more energy-dense formula (29.0% vs. 55.4%, *p* < 0.001), a protein supplement (27.6% vs. 56.3%, *p* < 0.001), and a prokinetic agent (38.7% vs. 48.8%, *p* = 0.03). There was no difference in the duration of feeding interruption (28 h vs. 30 h, *p* = 0.60). Implementation of feeding protocol did not affect ICU mortality (OR 0.508, CI 0.250–1.032, *p* = 0.06). The mortality predictors were SOFA score, underweight, and illness-related FI episode.

**Conclusion:**

Implementation of the feeding protocol improved feeding strategies and overall nutritional intake; however, it did not improve FI. Illness-related FI was associated with a reduction in survival of critically ill patients.

## 1 Introduction

The importance of nutrition therapy for critically ill patients is now emphasized in the medical community. Most critically ill patients admitted to the intensive care unit (ICU) are at risk of malnutrition, as they are usually associated with a hypercatabolic state and an increased energy requirement (1). The metabolism changes, energy expenditure, and nitrogen losses in ICU patients appear to vary over time (1, 2). In order to achieve adequate nutrition, enteral nutrition (EN) is now recommended as the first-line mode of feeding in critically ill patients with a functional gut but a compromised intake (3–5). Proper timing and optimal dosing of nutrition therapy play an important role in recovery from critical illness (3–7).

The most recent ICU nutrition guideline recommends that EN should be started within 24–48 h after admission, and to aim for 70% full feeding by day 3 of feeding implementation (8, 9). However, the energy requirements of critically ill patients are far from being met, despite the early initiation of enteral feeding (10). Few international observational studies have reported that 37–68% of patients did not meet their energy and protein requirements during their stay in the ICU (11, 12). Early and sufficient delivery of proteins as well as calories in ICU patients had impacts on clinically relevant outcomes such as longer ventilator-free days, reduction of financial cost, ICU and hospital length of stay (LOS), as well as duration of wound healing, decreased incidence of nosocomial infections, and mortality (5, 10, 12–16).

Among the reasons for inadequate nutrient delivery in critically ill patients, feeding interruption is highly prevalent (4, 17). It was found that the most common reason for EN feeding interruption is due to procedures (45.1%), followed by high gastric residual volume, GRV (38%), diarrhea (8.4%), difficulty in nasogastric tube placement (5.6%), and vomiting (2.9%) (18). In 2018, a study by Lee et al. categorized the causes of feeding interruption (FI) in the ICU into five main groups, which include procedure-related, illness-related, and gastrointestinal-related intolerance, as well as avoidable and unknown causes. The study reported that the occurrence of FI in the ICU is mainly due to human factors, either procedure-related and avoidable reasons, which contributed to a 3.6 times longer duration of feeding interruption than due to feeding intolerance (4).

Some of these factors can be improved with enteral feeding protocols to prevent underfeeding of critically ill patients and help achieve the targeted calorie goal. Barr et al. studied the application of evidence-based nutrition management protocol in the medical-surgical ICU, and they found that there was an increased likelihood that ICU patients who received EN protocols shortened their duration of mechanical ventilation (14). A few studies also showed that the use of a clear and concise evidence-based protocol has the advantage of reducing practice variations and ensuring standardization of care, leading to the improvement of patient care (14, 15, 17).

**Figure 1:**
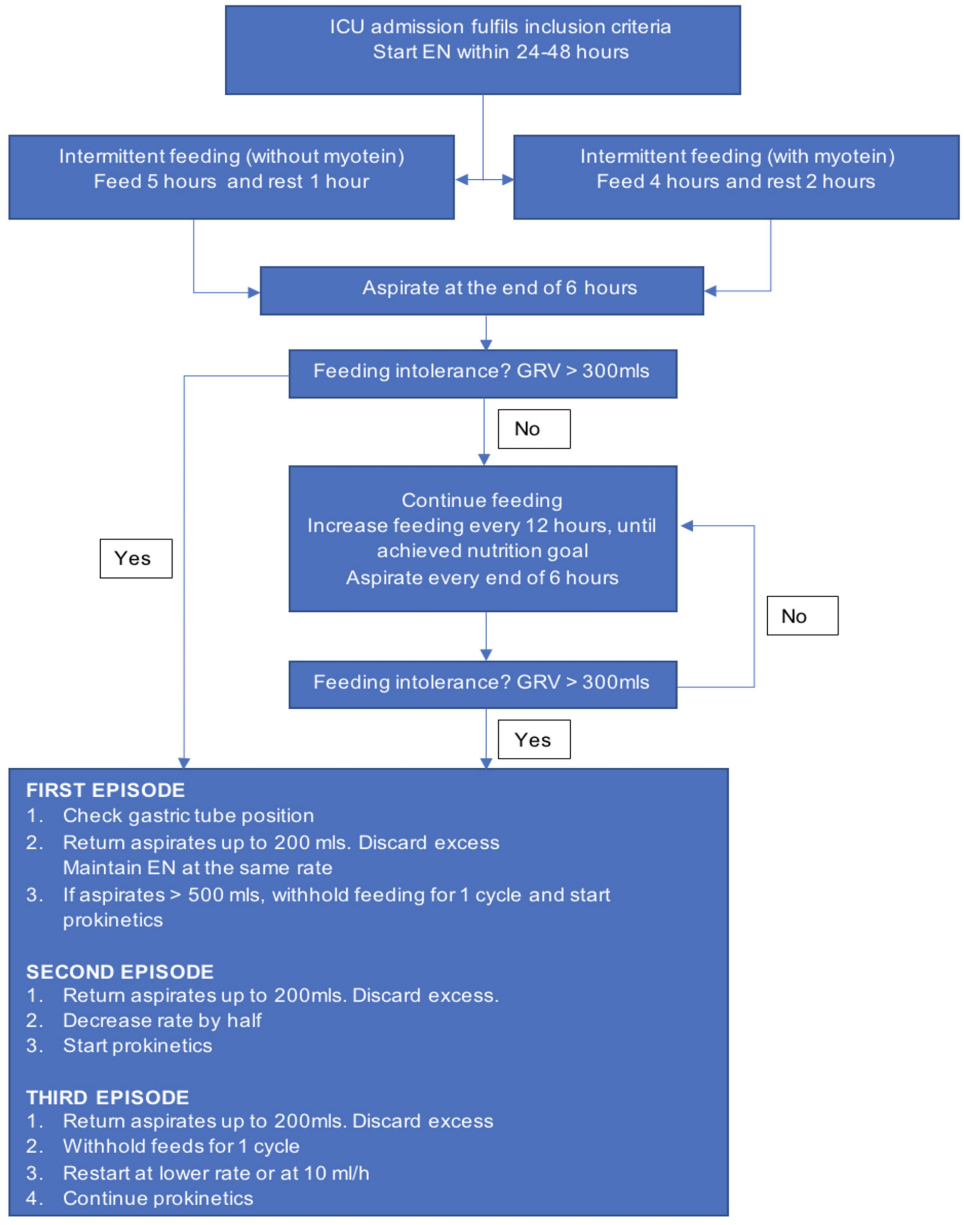
ICU feeding protocol flowchart and management of high GRV.

Regular training of staff who are involved in decision-making, implementation of the feeding process with the latest evidence-based feeding recommendations, and contemporary revision of existing feeding protocols are among the ways to minimize human-related FI. Our local protocol, as shown in Figure 1, was based on an intermittent feeding method that incorporated our enteral nutrition guideline (8). Despite the implementation of the enteral feeding protocol in our unit, the management of GRV, the process of increasing the rate of feeding, and the stoppage of feeding for pre-procedure differ among nurses and doctors. These contributed to interruption of feeding and delay in targeted calorie and protein intake. Therefore, in the present study, our objectives were to evaluate the effectiveness of the feeding protocol in improving the feeding interruption, nutrition adequacy, and clinical outcomes.

## 2 Materials and methods

### 2.1 Study design and ethical considerations

This was a single-center, retrospective, and prospective cohort study design with feeding protocol education intervention performed in the ICU of Hospital Canselor Tuanku Muhirz (HCTM). The study was approved by the Research Committee of the Department of Anaesthesiology & Intensive Care, Faculty of Medicine, and the Medical Research & Ethics Committee, Universiti Kebangsaan Malaysia (UKM) (JEP-825-2020). Convenient sampling was adopted during data collection. Electronic informed and signed consent was obtained from the healthcare staff who were recruited during the educational period of the study. Data collection was conducted by a single investigator along with her assistant.

### 2.2 Study population and data collection

The framework of this research included data collection before and after the introduction of education on the feeding protocol. The study comprised three phases. The first phase was a pre-protocol group, which involved patients who were admitted to the ICU between January 2020 and January 2021 before the implementation of the feeding protocol. The retrospective data collection for pre-protocol implementation was performed in the Medical Record Department, UKMMC. The second phase was the educational phase, which consisted of education based on videos, tutorials, and posters. The participants include anesthesia medical officer, registrar, specialist, ICU consultants, and nurses who work in the General ICU (GICU) and COVID-19 ICU. The duration of the education phase was 4 weeks. A link of the webpage for the educational video was distributed during the educational period, and an online lecture was performed by the investigator. A test was performed to ensure all participants were exposed and adhered to the standardized EN protocol. The feeding protocol was made available in the ICU throughout the period of study. The final phase was a post-protocol group that included prospective data collection for patients who were admitted between March 2021 and December 2021 after education on the EN protocol. Patients were recruited from GICU and COVID-19 ICU, which included those aged above 18 years, who received EN for more than 24 h, stayed in ICU for more than 48 h, and were mechanically ventilated within 48 h of ICU admission. Exclusion criteria included those who were pregnant, patients who were started on parenteral nutrition either as total or supplemental feeding, patients who had a previous ICU stay within the same hospitalization, and moribund patients who were severely ill with multi-organ failure, or not expected to survive within 48 h of ICU admission.

### 2.3 Enteral feeding protocol

Our ICU EN protocol is intermittent feeding for a total of 20 h a day, which includes 5 h of feeds and 1 h of rest for four cycles. The addition of protein supplementation (myotein) in the feeds reduced the total feeding duration to 16 h with 4 h of feeds and 2 h of rest. The standard, diabetic, and elemental formula with 1 kcaL/mL and an energy-dense formula with 2 kcaL/mL energy density were used in our ICU. Gastric residual volume was aspirated every time before the next feeding. High GRV was defined as gastric aspirates of more than 300 mL, and its management followed the workflow as shown in Figure 1 (8). Feeding interruption (FI) was defined as the omission of feeding for one cycle during the time when the patient should be given the intermittent feeds.

The energy requirement was targeted at 25 kcal/kg/day. As for protein requirement, it is targeted at 1.5 g/kg/day for patients with renal failure who did not require dialysis and 2 g/kg/day for those who require dialysis, as well as for those who do not have any kidney injury (8). Body mass index (BMI) was taken as calculation of weight, which was the weight of a person in kilograms divided by the square of their height in meters (kg/m^2^). The energy and protein intake were calculated using actual body weight in patients with normal BMI, which ranged from 18.5 to 24.9 kg/m^2^. Ideal body weight at a BMI of 22.5 kg/m^2^ was used for energy intake calculation for overweight patients who have a BMI ranging from 25 to 29.9 kg/m^2^. Adjusted body weight was used for those who were underweight (BMI less than 18.5 kg/m^2^) and obese (BMI at 30 kg/m^2^ or more), which is a 25% correction at 22.5 kg/m^2^ of BMI. The formulas for energy or protein adequacy, daily balance, cumulative balance, and deficit due to feeding interruption are as follows (4):


$$\;\text{Energy or protein adequacy}=\frac{\begin{array}{c} \mathrm{Sum}\;\text{of percentage}\;(\%)\;\\ \text{energy or protein received each}\;\mathrm{day} \end{array}}{\text{Total number of nutrition days}}$$

Energy or protein balance=Daily requirement–Daiy Intake.

$$\begin{array}{l} \text{Cumulative energy or protein balance}=\text{Total}\;\mathrm{sum}\;\text{of energy or}\;\\ \text{protein balance for nutrition days}. \end{array}$$

$$\begin{array}{l} \text{Energy or protein deficit}\;\mathrm{due}\;\text{to feeding interruption}=\text{Total}\;\mathrm{sum}\;\\ \text{of energy or protein balance during days of feeding interruption}. \end{array}$$

### 2.4 Study variables measurements

The demographic data of age, gender, height, weight, and BMI were recorded. ICU scores of Acute Physiology and Chronic Health Evaluation II (APACHE II) as well as Sequential Organ Failure Assessment (SOFA) were also charted. The clinical characteristics including the co-morbidities, COVID-19 status, number and duration of vasopressors, the length of mechanical ventilation, ICU stay, and mortality during ICU stay were also recorded.

The nutritional data was collected over 12 nutritional days. Nutritional days were defined as the length of ICU nutrition follow-up until death, discharge, or transition to oral feeding, whichever occurred earlier. The patient was considered a dropout if parenteral nutrition is started during the nutritional days and/or during ICU stay.

The nutritional risk status was assessed and collected using the modified-NUTRIC score. The score ranges from zero to nine, and a score of more than 5 is considered a high risk of malnutrition (2, 19). Permission for all the scoring systems used in the study was obtained from the author. Apart from that, the other nutritional risk parameters, including total nutrition days, timing to first EN, energy and protein requirement, intake, percentage of adequacy, as well as cumulative balance, were recorded. The efficiency of nutritional adequacy was analyzed from day 1 to day 7. Data on feeding interruption, including the cause, episodes, duration, and energy, as well as protein deficit, were charted. The cause of FI was categorized as follows for analysis:

1) Gastrointestinal (GI) related FI includes vomiting, diarrhea, feeding intolerance, or GI bleed.
2) Procedure-related FI includes radiological procedure, prone ventilation, any bedside procedure, feeding tube–related procedures, bronchoscopy, airway procedures, such as intubation/extubation, or operating theatre (OT) procedures.
3) Illness-related procedures such as hemodynamic or respiratory instability.
4) Avoidable causes such as unknown fasting reasons.

### 2.5 Enteral feeding protocol outcome

The primary outcome of our study was the evaluation of causes affecting ICU mortality. The secondary outcomes included assessment of risk factors affecting or associated with FI, ICU mortality, ICU length of stay, duration of mechanical ventilation, and duration of vasopressor requirement.

### 2.6 Statistical analysis

In total, 211 patients per arm were required to provide 80% power and *α* error of 0.05 to detect a 15% difference in the percentage of patients who reached caloric goals before and after enteral feeding protocol implementation, using the Fleiss formula (17). Data were analyzed using SPSS version 26.0 software package. Descriptive analysis was used to analyze the incidence of feeding interruption and clinical outcomes before and after the implementation of the feeding protocol.

Pertaining to the patient characteristics, continuous variables were conveyed as means ± standard deviation (SD) or median [Q1– Q3], while discrete variables were conveyed as counts (percentage, %). For data with normal distribution, differences between groups were calculated using the Student’s *t*-test. Skewed data were compared using the Mann–Whitney U test. Comparisons of categorical variables were performed using the Chi-square test or Fisher's exact test. Baseline characteristics that demonstrated significance between groups, *p-*value < 0.05, were further included as covariates in multivariable regression models in order to evaluate the independent effect of protocol implementation on nutritional outcomes. Friedman test and Wilcoxon Signed Rank test (*z*) were used to analyze the significance of energy adequacy from day 1 to day 7.

We used Spearman’s Rank Order Correlation test to measure the strength and direction of association between the duration of feeding interruption and ICU outcomes. The correlation strength was described as weak when the absolute value was below 0.50, moderate between 0.50 and 0.75, and strong with a value above 0.75. Logistic regression analysis was performed to identify the independent predictors of clinical outcome, which includes duration of ICU stays, mechanical ventilation, and ICU mortality. All the risk factors and other relevant variables with a *p-*value less than 0.2 in univariate analysis were entered stepwise into a multi-logistic regression model. The coupling variables were added and removed with a stepwise approach to obtain the final optimal model for factors predicting ICU mortality. A two-sided *p-*value of 0.05 or less was considered statistically significant.

### 2.7 Role of the funding source

The authors receive any specific grant from funding agencies in the public, commercial, or any other sectors for the conduct of this research. Publication of this article was supported by publication grant from the Faculty of Medicine, UKM.

## 3 Result

### 3.1 Demographic data and patients’ clinical characteristics

A total of 430 patients were recruited into the study, with 217 patients in the pre-protocol group and 213 patients in the post-protocol group. The comparison of demographic data and patients’ clinical characteristics are summarized in Table 1. The median age of patients in the pre-protocol group was significantly greater than the post-protocol group. A significantly higher number of medically based patients in the post-protocol group were admitted to the ICU. Among these patients, 76.9% were due to severe COVID-19 pneumonia. The mean age for patients with COVID-19 infection was 52 ± 14.5. The post-protocol group has a larger proportion of patients with acute respiratory distress syndrome (ARDS) upon ICU admission, 51.6% versus 28.1% in the pre-protocol group, with *p-*value <0.001, and among these patients, 72.6% were diagnosed with COVID-19.

**Table 1:** Demographic data and clinical characteristics.

Demographic data	Pre-protocol (*n* = 217)	Post-protocol (*n* = 213)	*p-*value
Age (year)	60 [46–68]	55 [38–65]	0.003*
Gender			
Male	142 (65.4)	121 (56.8)	0.06
Female	75 (34.6)	92 (43.2)	
Covid status			
Covid	0 (0)	135 (63.4)	<0.001*
Non-Covid	217 (100)	78 (36.6)	
Type of admission			
Medical based	110 (50.7)	173 (81.2)	<0.001*
Surgical based	103 (49.3)	40 (18.8)	
Comorbidity			
Hypertension	135 (62.2)	118 (55.4)	0.15
Diabetes Mellitus	105 (48.4)	86 (40.4)	0.09
Lung disease	26 (12)	20 (9.4)	0.38
Heart disease	61 (28.1)	30 (14.1)	<0.001*
Liver disease	10 (4.6)	6 (2.8)	0.32
Kidney disease	51 (23.5)	25 (11.7)	0.001*
Malignancy	19 (8.8)	12 (5.6)	0.21
Smoker	16 (7.4)	21 (9.9)	0.35
Obesity	34 (15.7)	74 (34.7)	<0.001*
Number of co-morbidities	3 [1–4]	2 [1–3]	<0.001*
Disease severity score			
APACHE	19 [14–24]	16 [12–21]	0.002*
SOFA	8 [5–10]	5 [4–7]	<0.001*
Clinical characteristics			
Anthropometric			
Height (cm)	163.3 ± 8.4	163.9 ± 7.7	0.4
Weight (kg)	67 [57–75]	72 [65–90]	<0.001*
BMI (kg/m^2^)	24.9 [21.7–28.3]	27.7 [23.9–33.3]	<0.001*
Modified NUTRIC score			
Low risk	98 (45.2)	143 (67.1)	0.004*
High risk	119 (54.8)	70 (32.9)	<0.001*
Clinical status			
Number of patients on dialysis	71 (32.7)	56 (26.3)	0.14
Number of patients in shock requiring vasopressors	178 (82)	152 (71.4)	0.009*
Number of vasopressors used	1 [1–1]	1 [0–1]	0.043*
Duration of vasopressors (hours)	40 [6.5–100.5]	38 [0–112]	0.52

Data are expressed in mean ± standard deviation, frequency (percentage), or median [Q1–Q3] as appropriate. BMI, body mass index; APACHE, Acute Physiology and Chronic Health Evaluation; SOFA, Sequential Organ Failure Assessment; NUTRIC, Nutrition Risk in Critically Ill; ICU, Intensive Care Unit; ARDS, Acute Respiratory Distress Syndrome. *p-value < 0.05.

Patients with heart and kidney disease co-morbidities were significantly larger in the pre-protocol group, while more obese and higher BMI patients were enrolled in the post-protocol group. Despite the higher percentage of kidney disease patients in the pre-protocol group, the requirement for dialysis was not significant between the groups. Patients in the pre-protocol group had a significantly larger number of co-morbidities, a higher disease severity score, risk of developing malnutrition, and the majority required vasopressors compared to the post-protocol group.

### 3.2 Adjusted analysis of baseline characteristics

To address the baseline differences in patients’ characteristics between the pre-protocol and post-protocol groups, multivariable linear regression analysis was conducted to evaluate the association between protocol implementations and nutritional outcomes. After adjusting for age, COVID-19 status, type of admission, BMI, heart and kidney disease, obesity, ARDS, disease severity scores, modified NUTRIC score, numbers of comorbidities, and vasopressors requirement, protocol implementation was associated with a significant increase in energy and protein adequacy (*β* = 15.55, 95% CI 10.68–20.43, *p-*value < 0.001 and *β* = 16.53, 95% CI 11.70–21.36, *p*-value < 0.001), earlier initiation of feeding (*β* = −10.10, 95% CI −19.37 to −0.84, *p*-value = 0.03), shorter duration of FI (*β* = −10.76, 95% CI −20.97 to −0.55, *p*-value = 0.04), and reduction in energy deficit (*β* = −615.06, 95% CI −1072.67 to −157.45, *p*-value = 0.009). Protocol implementation did not contribute to the reduction in protein deficit (*p*-value = 0.09), after adjusting for demographic confounders.

### 3.3 Nutritional characteristics

The summary of the nutritional characteristics is shown in Table 2. The total evaluable EN days were 3,670 days, with a median of 7 [5–11] days pre-protocol and 11 [7–17] days post-protocol*, p-*value < 0.001. Enteral feeding was initiated earlier after implementation of the protocol. A total of 71.9% of patients in pre-protocol received their first feeding within 24 h of ICU admission in comparison with 79.8% of patients, *p*-value = 0.011. The preference for types of EN formula administered had significantly changed from the standard formula in the pre-protocol group to the energy-dense formula after implementation of the protocol. A larger number of post-protocol patients received extra protein in the feeding. The use of prokinetic agents was also increased in the post-protocol group.

**Table 2:** Nutritional characteristics between the pre- and post-protocol groups.

Nutritional characteristics	Pre-protocol (*n* = 217)	Post-protocol (*n* = 213)	*p-*value
Total EN (day)	7 [5–11]	11 [7–17]	<0.001*
Time from admission to EN (hours)	12 [4.5–11.0]	10 [1.0–24.0]	<0.001*
Feeding formula			
Standard formula	98 (45.2)	45 (21.1)	<0.001*
Diabetic formula	55 (25.3)	48 (22.5)	0.49
Elemental formula	1 (0.5)	2 (0.9)	0.56
Energy-dense formula	63 (29.0)	118 (55.4)	<0.001*
Addition of protein powder in feeding	60 (27.6)	120 (56.3)	<0.001*
Use of prokinetic	84 (38.7)	104 (48.8)	0.03*
Erythromycin	34 (15.7)	58 (27.2)	0.03*
Metoclopromide	81 (37.3)	103 (48.4)	0.02*
FI			
Total day with FI	3 [2–5]	3 [2–5]	0.12
Total duration of FI, hours	28 [18.0–50.5]	30 [14.0–54.5]	0.60
Average energy deficit (kcal)	547.7 [385.3–822.8]	596 [376.7–824.8]	0.87
Average protein deficit (gram)	24.23 [16.3–35.8]	28.1 [16.9–42.1]	0.06
Energy (kcal)			
Calculated energy intake	8,500 [4100–12,500]	13,000 [10000–15,520]	<0.001*
Actual energy intake	5781.2 [2335–9651.3]	10,450 [6700–13,525]	<0.001*
Cumulative energy balance	1927 [1093–3069.2]	1824 [867.7–3,180]	0.27
Energy intake adequacy, %	67.3 [52.5–80.4]	79.9 [65.1–88.8]	<0.001*
Protein (gram)			
Calculated protein intake	360.8 [397.50]	649.5 [290.38]	<0.001*
Actual protein intake	247.72 [337.56]	529 [366.22]	<0.001*
Cumulative protein balance	84.59 [52.5–80.4]	84.06 [114.91]	0.75
Protein intake adequacy, %	67.3 [52.3–80.4]	80 [68.3–88.9]	<0.001*

Data are expressed in mean ± standard deviation, frequency (percentage), or median [Q1–Q3], as appropriate. EN, enteral feeding; FI, feeding interruption. *p-value < 0.05.

The total energy and protein intake, as well as adequacy, were significantly greater after protocol implementation. The planned energy and the actual energy intake per patient (kcal/kg/day) in the post-protocol were significantly increased compared to the pre-protocol group, 20.6 [17.4–23.8] vs. 18 [14.4–22.4], *p-*value < 0.001, and 17 [13.5–20.7] vs. 12 [8.1–17.1], *p-*value < 0.001, respectively. The timing of EN initiation from admission contributed to the energy and protein adequacy (*β* = 18.93, 95% CI 15.13–22.74, *p*-value < 0.001, and *β* = 19.52, 95% CI 15.75–23.30, *p*-value < 0.001, respectively). The addition of protein in EN also aids in energy and protein adequacy (*β* = 7.86, 95% CI 4.51–11.20, *p*-value < 0.001 and *β* = 7.76, 95% CI 4.44–11.08, *p*-value < 0.001). Energy-dense formula and use of prokinetic did not add to the energy and protein adequacy despite a higher prescription in the post-protocol group. The energy-dense formula also did not contribute to energy and protein adequacy in comparison to the standard formula. The cumulative energy and protein balance were similar in both groups.

The cumulative energy deficit is comparable in both groups, 1927.5 [1093.0–3069.2] in pre-protocol versus 1824.0 [867.7–3180.0] in post-protocol group, *p*-value = 0.28. It was observed that the interaction between protocol and FI demonstrated a higher energy deficit of 28.12 kcal for every hour of FI in the post-protocol group than the pre-protocol group (*β* = 28.12, 95% CI 22.65–33.59, *p*-value < 0.001). Energy deficit was also increased with a greater delay in EN feeding initiation (*β* = 5.78, 95% CI 1.24–10.33, *p-*value < 0.001) and when a higher number of vasopressor support was prescribed (*β* = 258.7, 95% CI 76.90–440.52, *p*-*value* = 0.005).

As for cumulative protein deficit, 84.6 [48.6–136.3] in pre-protocol versus 84.1 [42.7–157.6] in post-protocol group, *p*-value = 0.76, was observed. The protein deficit is greater in the post-protocol group when FI occurs (*β* = 1.50, 95% CI 1.19–1.80, *p-*value < 0.001). Protein deficit is also higher when patients receive more vasopressor (*β* = 14.78, 95% CI 4.62–24.94, *p*-value = 0.004) and whenever there is a delay in EN feeding (*β* = 0.30, 95% CI 0.04–0.55, *p*-value = 0.02).

**Figure 2:**
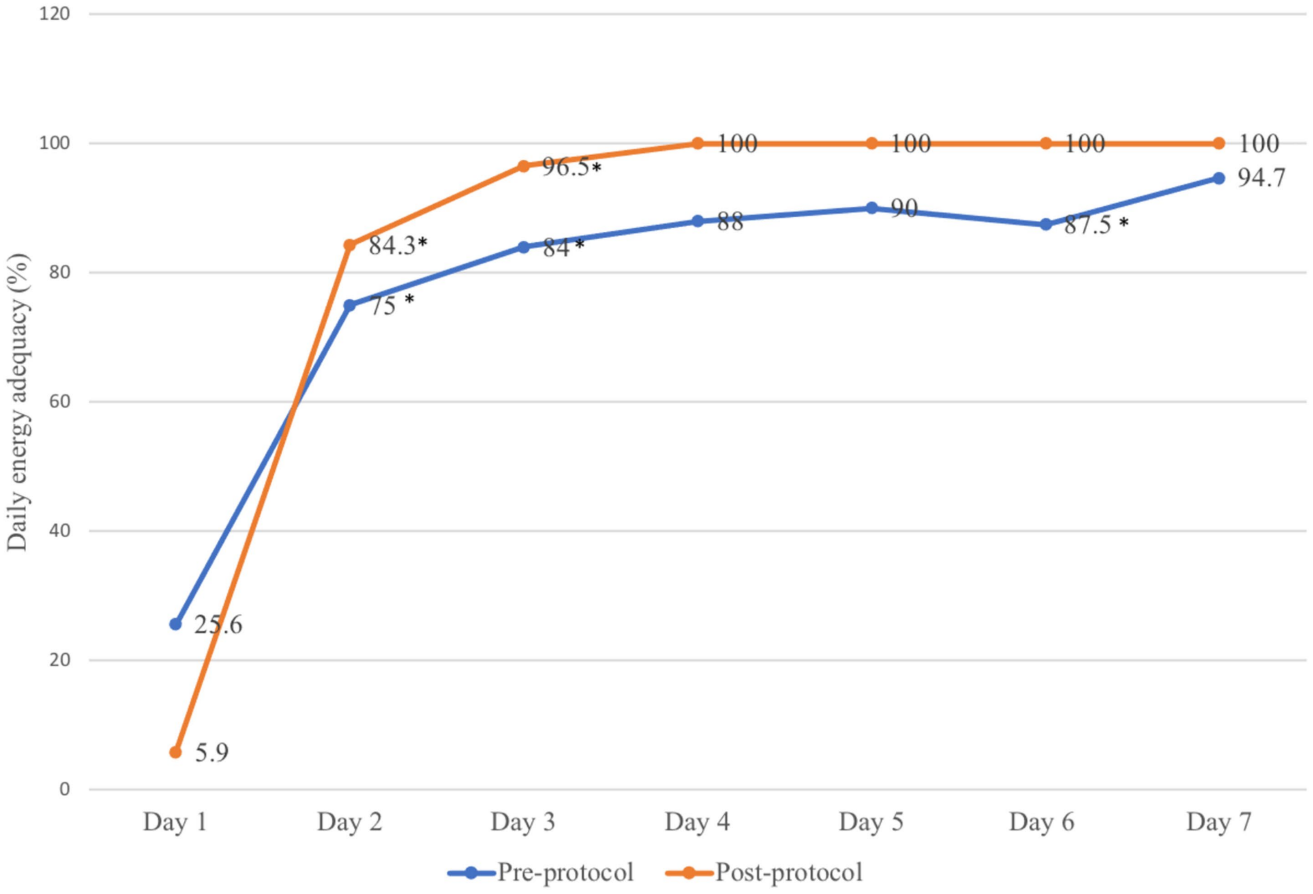
Daily energy adequacy provided before and after implementation of the feeding protocol. *p-value < 0.05.

Figure 2 shows the daily energy adequacy of both groups. In the pre-protocol group, the energy adequacy increased from 25.6% on day 1 to 75% on day 2 (*p-*value < 0.001), from day 2 to day 3 (75% vs. 84%, *p*-value = 0.001) with slight decrement of feeding rate between day 5 to day 6 (90 to 87.5%, *p*-value = 0.03). As for post-protocol group, the energy adequacy significantly raised from 5.9% in day 1 to 84.3% in day 2 (*p*-value < 0.001) and day 2 to day 3 (84.3% vs. 96.5%, *p-*value < 0.001). The post-protocol group also achieved full feeding faster on day 4 onwards than the pre-protocol group.

Comparison between intergroup demonstrated that the type of admission, APACHE II, and SOFA scores were not significant when compared with nutritional outcomes. However, patients with high NUTRIC score (*n* = 189) experienced slower feeding initiation, 13.0 [4.0–28.0] versus 8.0 [1.0–24.0], *p*-value = 0.006, needed frequent and longer vasopressor administration (53.0% vs. 14.0%, *p-*value < 0.001 and 74.0 [34–141.5] vs. 14.0 [0.0–54.5], *p-*value < 0.001), in comparison with low NUTRIC score patients. Among patients receiving vasopressors (*n* = 330), timing for EN was significantly delayed compared to those not on vasopressors, 12.0 [3.0–27.0] versus 6.5 [0.0–18.0], *p*-value = 0.01. Energy and protein adequacy were also significantly lower in the vasopressor group [72.7 (58.1–84.0) vs. 80.5 (62.6–89.5), *p*-value = 0.007, and 73 (57.3–84.1) versus 80.5 (63.9–89.6), *p*-value 0.003], respectively. In addition, the energy and protein cumulative deficit were significantly greater in those receiving vasopressors [2001.0 (1096.5–3457.8) vs. 1447.1 (747.8–2150.0), *p-*value < 0.001, and 92.4 (48.9–161.3) vs. 62.3 (31.9–97.2), *p-*value < 0.001], correspondingly. It was observed that dialysis patients (*n* = 127) receives longer duration of vasopressor [98.0 (24.0–217.0) vs. 29.0 (0.0–69.0), *p-*value < 0.001], delayed initiation of EN [16.0 (4.0–36.0) vs. 9.0 (2.0–24.0), *p*-value = 0.001], higher energy deficit [2446.0 (1360.0–4100.0) vs. 1650.0 (925.0–2834.4), *p-*value < 0.001] as well as protein deficit [112.5 (58.9–199.9) vs. 76.5 (42.7–132.0), *p-*value < 0.001] compared to non-dialysis patients.

### 3.4 Comparison of prevalence and causes of feeding interruption

A total of 12 patients (5.6%) in the post-protocol group had inappropriate cessations of feeding. The prevalence of FI remained high despite implementation of the feeding protocol (98.2% in pre-protocol vs. 97.2% in post-protocol group). The pre-protocol FI was noted to be less frequent when comparing the frequency of FI in the post-protocol group, 789 versus 863 episodes. The total duration of FI of all causes was 8,987 h, with an average of 11.4 h in each episode of FI for the pre-protocol group, and 9,120 h in the post-protocol group, with an average 10.6 h per episode of FI. The difference in median time for FI in both groups was not statistically significant, 28 [18–50.5] hours in the pre-protocol versus 30 [14–54.5] hours in the post-protocol group, *p*-value = 0.60. The number of FI days was also similar in both groups [3 (2–5) in pre-protocol vs. 3 (2–5) in post-protocol group, *p*-value = 0.12]. The energy and protein deficit during FI were comparable, as shown in Table 2.

For procedure-related FI, which includes radiological, extubation and intubation, OT, and bedside procedure, the duration was significantly shorter in the post-protocol than the pre-protocol group. The duration of fasting and the unknown cause of FI were also significantly less in the post-protocol group. Prone ventilation and the duration of FI as a result of respiratory instability were significantly longer in the post-protocol group in comparison with the pre-protocol group, as shown in Table 3. The subgroup analysis of FI duration related to hemodynamic instability and GI, which includes FI due to vomiting, diarrhea, high gastric aspirate, and GI bleeding, was comparable between groups.

**Table 3:** Comparisons of FI duration subgroup between pre-protocol and post-protocol.

Duration of FI (hours)	Pre-protocol (*n* = 217)	Post-protocol (*n* = 213)	*p*-value
Procedure-related FI			
Radiological procedure	0.0 [0.0–3.5], range 0–66	0.0 [0.0–0.0], range 0–24	<0.001
Extubation/intubation	5.0 [0.0–13.0], range 0–48	1.0 [0.0–8.0], range 0–42	0.002
OT procedure	0.0 [0.0–0.0], range 0–40	0.0 [0.0–3.0], range 0–36	< 0.001
Bedside procedure	0.0 [0.0–0.0], range 0–14	0.0 [0.0–0.0], range 0–0	0.003
Prone ventilation	0.0 [0.0–0.0], range 0–0	0.0 [0.0–4.0], range 0–75	< 0.001
Illness related FI			
Respiratory instability	0.0 [0.0–0.0], range 0–56	0.0 [0.0–0.0], range 0–264	< 0.001
Avoidable cause			
Unknown cause	0.0 [0.0–0.0], range 0–55	0.0 [0.0–0.0], range 0–0	< 0.001
Fasting of undetermined reasons	0.0 [0.0–4.0], range 0–316	0.0 [0.0–0.0], range 0–48	<0.005

Data are expressed in median [Q1-Q3] and range as appropriate.

In comparison to the standard formula, the total duration of FI is longer in patients receiving the energy-dense formula [38.0 (17.0–68.5) vs. 24.0 (16.8–42.3), *p*-value = 0.004]. This difference was contributed by FI related to respiratory instability [0 (0–0), range 0–264 vs. 0 (0–0), range 0–132, *p*-value = 0.001], GI bleeding [0 (0–0), range 0–168 vs. 0 (0–0), range 0–96, *p*-value = 0.04], prone position [0 (0–0), range 0–75 vs. 0 (0–0), range 0–48, *p-*value < 0.001] and fasting [0 (0–0), range 0–132 vs. 0 (0–4), range 0–92, *p*-value = 0.02]. Furthermore, the energy and protein deficit in energy-dense formula is higher in comparison to standard formula [2,504 (1418–3,907) vs. 1,675 (1060–2,517), *p-*value < 0.001, and 122.5 (67.5–199.5) vs. 69.6 (45.2–104.9), *p-*value < 0.001].

**Figure 3:**
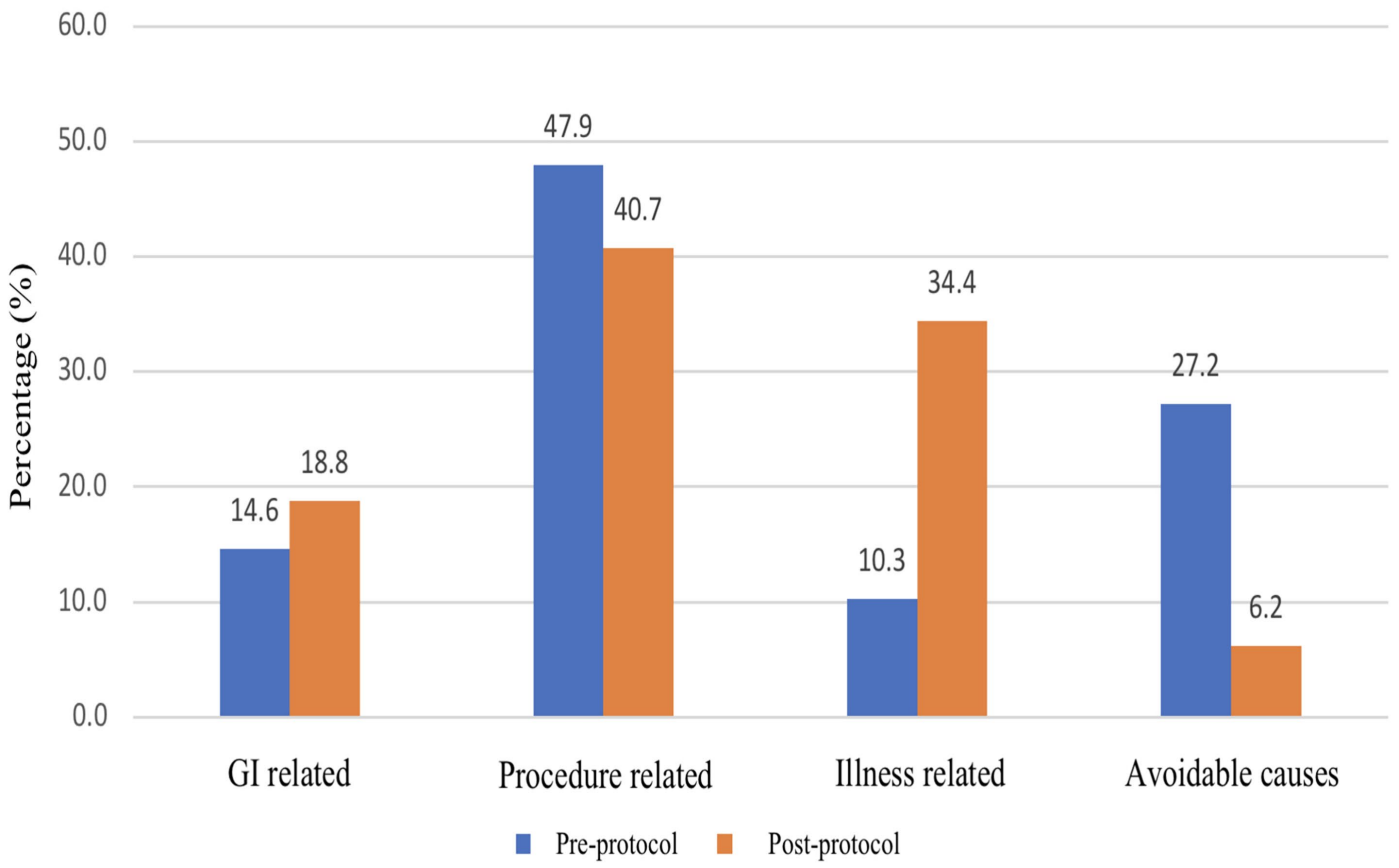
Prevalence of different causes of feeding interruption between pre-protocol and post-protocol groups.

**Figure 4:**
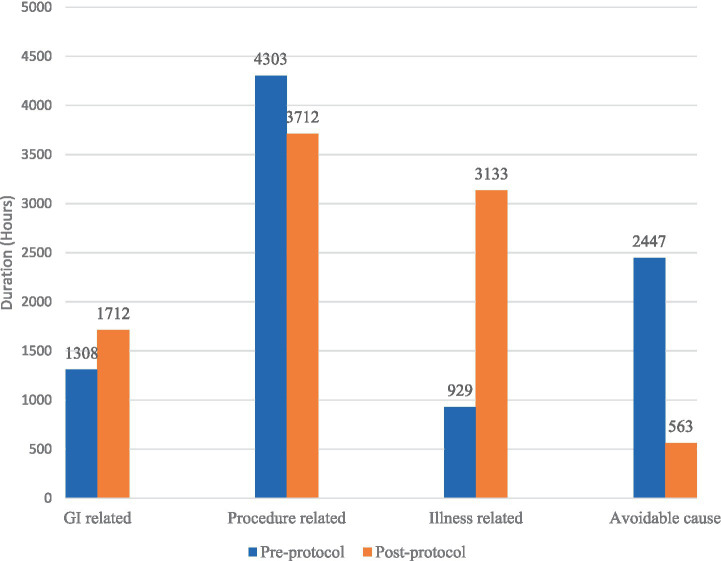
Duration of different causes of feeding interruption between pre-protocol and post-protocol groups.

The common cause of FI in the pre-protocol group was due to extubation and intubation procedures, with a prevalence of 63.6 and 22.7% of total episodes of FI. The longest duration of FI in pre-protocol was due to fasting of undetermined reasons, with a duration of 1920 h, which comprises 21.4% of the total FI duration. On the other hand, the highest prevalence of FI in the post-protocol group was also due to extubation and intubation procedures, which occurred in 52.1% of cases. The greatest episodes of FI were due to the radiology procedure that occurred in 23.5% of patients. The longest duration of FI was caused by respiratory instability, with a duration of 2,517 h that constitutes 27.6% of the total duration of FI. After implementation of the feeding protocol, there were no reported FI episodes that were related to unknown reasons in comparison to 3.8% of cases in the pre-protocol group. Figures 3, 4 summarize the prevalence and duration of different categories of FI pre- and post-protocol group.

### 3.5 Outcomes and its predictors

The duration of ICU stays and mechanical ventilation were significantly longer in post-protocol group 8.0 [5.0–12.0] days versus 12.0 [8.0–18.0] days, *p-*value < 0.001, and 6.0 [4.0–10.0] days versus 11.0 [5.0–17.0] days, *p-*value < 0.001, respectively. Protocol implementation and COVID-19 infection did not affect the outcome in the duration of ICU stays and mechanical ventilation. Late initiation of feeding (AOR 0.03, 95% CI 0.02–0.04, *p*-value < 0.001), longer duration of vasopressor use (AOR 0.005, 95% CI 0.000–0.009, *p-*value = 0.31), and mechanical ventilation (AOR 0.66, 95% CI 0.60–0.72, *p*-value < 0.001) accord with the greater length of ICU stays. The duration of mechanical ventilation increases when the duration of FI caused by diarrhea was longer (AOR 6.47, 95% CI 1.78–11.16, *p-*value = 0.007), with a higher use of prokinetic (AOR 3.08, 95% CI 1.67–4.48, *p*-value < 0.001), a longer duration of vasopressor (AOR 0.03, 95% CI 0.02–0.04, *p*-value < 0.001), and in obese patients (AOR 2.82, 95% CI 0.46–5.20, *p*-value = 0.19). The duration of vasopressor requirement was not significantly different between the pre-protocol group, 40.0 [6.5–100.5] hours, and 38.0 [0.0–112.0] hours in the post-protocol group.

The mortality in the post-protocol group was significantly higher than the pre-protocol group (42.7% vs. 24.4%, *p-*value < 0.001). Protocol implementation was not associated with mortality outcome (*p*-value = 0.58). COVID-19 patients demonstrated the strongest factor for mortality with an adjusted odds ratio of 31.70 (95% CI 8.53–117.58, *p-*value < 0.001). Longer duration of FI in COVID-19 patients was positively correlated with average energy deficit, ICU stay, vasopressor, and mechanical ventilation duration (R_s_ = 0.264, *p*-value *=* 0.002; R_s_ = 0.244, *p*-value *=* 0.04; R_s_ = 0.337, *p-*value < 0.001 and R_s_ = 0.179, *p*-value *=* 0.04, respectively).

### 3.6 Subgroup analysis of non-COVID-19 patients

After exclusion of COVID-19 patients, it was observed that no significant differences between pre-protocol (*n* = 217) and post-protocol (*n* = 78) groups pertained to age, APACHE II, or NUTRIC scores, although post-protocol patients were younger, 60 [46–68] versus 56 [38–66], *p*-value 0.06. Compared with the pre-protocol group, post-protocol patients had lower rates of heart disease (15.4% versus 28.1%, *p*-value = 0.025), kidney disease (7.7% versus 23.5%, p-value = 0.002) and dialysis use (16.7% versus 32.7%, *p*-value = 0.007) but higher incidences of obesity (26.9% versus 15.7%, *p*-value = 0.029) and smoking (16.7% versus 7.4%, p-value = 0.018). The post-protocol group also had a significantly fewer co-morbidities [2 (1–3) versus 3 (1–4), *p*-value = 0.019], lower SOFA scores [5.5 (4–9) versus 8 (5–10), *p*-value = 0.003], less frequent use as well as reduced number of vasopressors [66.7% versus 82.0%, *p*-value = 0.05 and 1 (0–1) versus 1 (1–1), *p*-value = 0.006].

In addition, the time of starting feeding is shorter [3.0 (0.0–15.3) versus 12.0 (4.5–11.0), *p-*value < 0.001], the energy and protein adequacy are higher [83.6 (74.8–91.8) versus 67.3 (52.5–80.4), *p*-value < 0.001] and [83.6 (76.6–91.2) versus 67.3 (52.3–80.4), *p*-value < 0.001], respectively, in the post-protocol than the pre-protocol group. The duration and total days of FI were also lesser in the post-protocol group, 19.5 [8.0–36.8] versus 28.0 [18.0–50.5], *p-*value < 0.001, and 2.0 [2.0–4.0] versus 3.0 [2.0–5.0], *p-*value < 0.001, respectively. Feeding interruption due to vomiting and radiological procedures occurred less frequently in the post-protocol group, [0 (0–0), range 0–20 versus 0 (0–0), range 0–40, *p*-value = 0.01 and 0 (0–0), range 0–9 versus 0 (0–3.5) range 0–66, *p*-value = 0.001], respectively. In contrast, FI related to prone positioning was longer, 0 [0–0], range 0–75 versus 0 [0–0], range 0–0, *p*-value = 0.018. No documented FI due to an unknown cause was observed in the post-protocol group, 0 [0–0], range 0–0 versus 0 [0–0], range 0–55, *p*-value = 0.004.

The cumulative energy and protein deficit was smaller in the post-protocol group than the pre-protocol group, 1192.0 [699.1–2047.5] versus 1927.5 [1093.0–3069.2], *p*-value < 0.001, and 53.7 [32.7–99.7] versus 84.6 [48.6–136.3], *p*-value < 0.001, respectively. The addition of myotein was also significant between groups (27.6% in pre-protocol versus 42.3% in post-protocol, *p*-value = 0.017). The use of prokinetics and different types of feeding formula was not significant between groups.

The length of ICU stays was longer in the post-protocol group in comparison to the pre-protocol group [11.0 (5.0–18.0) versus 8.0 (5.0–12.0), *p-*value < 0.001]. Higher BMI (AOR 0.10, 95% CI 0.01–0.187, *p*-value = 0.02), delayed enteral feeding (AOR 0.02, 95% CI 0.004–0.042, *p*-value = 0.02), longer duration of vasopressor use (AOR 0.006, 95% CI 0.001–0.012, *p*-value = 0.03), and mechanical ventilation (AOR 0.58, 95% CI 0.50–0.642, *p-*value < 0.001) were associated with greater duration of ICU stays. As for the duration of mechanical ventilation, patients in the post-protocol group were ventilated longer than the pre-protocol group [6.0 (4.0–10.0) versus 10.5 (5.0–17.0), *p-*value < 0.001]. It was observed that an extended period of FI due to diarrhea (AOR 6.0, 95% CI 1.63–10.27, *p*-value = 0.007), more prokinetic use (AOR 4.13, 95% CI 2.52–5.75, *p-*value < 0.001) and longer duration of vasopressor (AOR 0.03, 95% CI 0.03–0.04, *p-*value < 0.001) contributed to the increased duration of mechanical ventilation.

The implementation of feeding protocol was not associated with an improvement in the length of ICU stays (AOR 1.28, 95% CI 0.03–2.60, *p*-value = 0.06), duration of mechanical ventilation (OR 1.26, 95% CI -1.07 to 3.59, *p*-value = 0.29) or mortality (OR 0.508, 95% CI 0.250–1.032, *p*-value = 0.06). However, the feeding protocol demonstrated improvement in energy (*β* = 7.46, 95% CI 1.95–12.96, *p*-value = 0.008) and protein adequacy (*β* = 11.56, 95% CI 7.20–15.91, *p-*value < 0.001) as well as earlier feeding initiation (*β* = −6.50, 95% CI -12.36 to 0.65, *p*-value = 0.03) in the post-protocol group. The interaction between feeding protocol and duration of FI showed that every hour of increase in FI occurs in the post-protocol group will increase the energy deficit by 33 kcal and 1.41 g in protein deficit (*β* = 33.0, 95% CI 20.1–45.8, *p-*value < 0.001) and (*β* = 1.41, 95% CI 0.80–2.01, *p-*value < 0.001), respectively. Age, APACHE and SOFA score, NUTRIC score, medical-based admission, underweight, duration of mechanical ventilation and vasopressor, duration of FI, illness-related FI episode, GI-related FI episode, and duration were identified as univariate factors affecting ICU mortality, as shown in Table 4.

**Table 4:** Univariate factors that affect ICU mortality.

Variable (*n* = 295)	OR (95% CI)	*p-*value
Age	1.022 (1.003–1.041)	0.020*
APACHE	1.057 (1.019–1.096)	0.003*
SOFA	1.298 (1.192–1.413)	<0.001*
NUTRIC score	3.477 (1.899–6.476)	<0.001*
Medical-based admission	1.995 (1.126–3.536)	0.018*
Underweight	6.884 (2.609–18.168)	<0.001*
Duration of vasopressor	1.006 (1.004–1.009)	<0.001*
Duration MV	1.056 (1.024–1.089)	<0.001*
Duration of FI	1.016 (1.008–1.024)	<0.001*
Illness related FI episode	4.902 (2.734–8.789)	<0.001*
GI-related FI episode	1.189 (1.006–1.404)	0.042*
GI-related FI duration	1.019 (1.004–1.035)	0.013*

OR, Odds Ratio; CI, Confidence Interval; APACHE, Acute Physiology and Chronic Health Evaluation; SOFA, Sequential Organ Failure Assessment; NUTRIC, Nutrition Risk in Critically Ill; MV, mechanical ventilation; FI, feeding interruption; GI, gastrointestinal. *p-value < 0.05.

The multivariate factors that predicted ICU mortality were SOFA score (AOR 1.263, 95% CI 1.123–1.421, *p-*value < 0.001), underweight (AOR 6.210, 95% CI 1.723–22.381, *p-*value = 0.005), and illness related FI episode (AOR 3.026, 95% CI 1.490–6.147, *p*-value = 0.002). Although the duration of FI was not an independent predictor for the length of ICU stays (*p*-value = 0.21), duration of mechanical ventilation (*p*-value = 0.50), and ICU mortality (AOR 1.004, 95% CI 0.993–1.016, *p*-value = 0.46), a significant positive correlation was observed between FI duration and the duration of ICU stays (R_s_ = 0.250, *p*-value < 0.001), duration of vasopressor (R_s_ = 0.345, *p-*value < 0.001), and mechanical ventilation (R_s_ = 0.209, *p-*value < 0.001).

## 4 Discussion

In this present study, we evaluate feeding interruption, nutrition adequacy, and clinical outcomes before and after implementation of the feeding protocol. Our study did not show any significant improvement in the frequency and total duration of FI, except among non-COVID-19 cohorts. The observed outcome may be attributed to the frequent FI in COVID-19 patients, as previously described by Liu et al., who highlighted that FI was commonly encountered in patients with COVID-19, likely due to higher illness severity and increased risk of gastrointestinal intolerance (20). In our study, we also observed that the majority of FI occurred mainly in COVID-19 patients. They tend to develop feeding intolerance due to impaired gut function, large GRV, and vomiting as a result of high vasopressor requirement, respiratory instability, medications, hyperglycemia, hypoxic event, or the disease process, as described by other studies (20, 21). In our ICU, COVID-19 with ARDS comprises 72.6%, and among these, 42.9% of patients required prone ventilation. The EN was withheld during prone ventilation as most ICU staff were concerned with the increased risk of feeding aspiration. In contrast to our practice, Raymond et al. in 2021 demonstrated that EN was well tolerated, without an increase in EN intolerance or complications for patients who were fed in the prone position (22). As such, the prone position did not preclude the use of EN, and the feeding should not be delayed. In 2021, Singer et al. suggested that a low-dose trophic feeding of 10 mL/h could be considered in the prone position with slow advancement in the feeding rate if there were no signs of enteral feeding intolerance (23), early use of prokinetics, followed by post-pyloric feeding if there was a persistent gastric retention (23–25). These strategies, if incorporated, may have mitigated the prolongation of FI in patients with respiratory instability and in those who required prone ventilation in the post-protocol group. However, the strategies were not included as the protocol was developed prior to the pandemic. It is also worth noting that these types of interruptions related to illness and GI in nature appear to be primarily driven by the severity of underlying illness rather than modifiable clinical practices or habitual care patterns. As such, protocol alone may not be sufficient to address interruptions caused by the progression of the disease.

It was also observed that the prevalence of FI in the critically ill patients remained high after implementation of the feeding protocol. A total of 1,550 days of FI were documented, which comprises 30% of nutritional days. These findings were relatively higher in comparison with Lee et al. (4) and Uozumi et al. (13), who demonstrated the prevalence of FI was 12.8 and 19% of the total number of evaluable nutrition days, respectively. Interestingly, although the overall frequency and total duration of FI remained high, our sub-analysis revealed a notable improvement in the duration of specific categories of FI, which include extubation or intubation and radiological procedures.

A greater number of FI cases were related to procedures involving extubation and intubation, which was consistent with other studies (4, 26, 27). Despite the fasting time pertaining to the extubation and intubation procedure, there was an improvement in the post-protocol period; however, it was not uncommon to see some of the patients fasting overnight before the procedure. A study by Pousman et al. (28) demonstrated that a shortened fasting protocol by aspiration of gastric feeds 45 min before extremity surgery in mechanically ventilated patients was safe, without significant vomiting complications, and showed improvement in nutrition delivery. Guidelines released by the Society of Critical Care Medicine and American Society for Parenteral and Enteral Nutrition also suggested that the status of nil by mouth in relation to procedure should be minimized in order to limit the development of ileus and prevent inadequate nutrient delivery (29).

It was also observed that FI due to radiological procedures in the pre-protocol group often occurred more than once daily, leading to fragmented and repeated feeding cessations that eventually contributed to the duration of FI. Our analysis showed that the duration of FI improves after the implementation of the feeding protocol. It is understood that the process of fasting for a radiology procedure is related to the prevention of vomiting that may occur as part of a reaction toward intravenous contrast, which in severe cases may lead to aspiration pneumonia. However, current evidence demonstrates that the incidence of vomiting caused by contrast is very minimal and the risk of aspiration pneumonia is almost nil (30, 31). A national survey on UK-based intensive care units concluded that a considerable variation of fasting period related to procedure was observed, which may lead to under-delivery of EN, and patients who were scheduled for radiology procedures had a shorter fasting time whenever fasting guidelines were in place (32).

Despite our observation of a similar duration of hemodynamic instability FI between groups, it is important to highlight that one of the reasons for a high prevalence of FI could still be due to withholding of EN in shock patients. A greater proportion of our patients presented with shock and required a higher number of vasopressors for hemodynamic stabilization. Consequently, EN initiation was delayed, resulting in lower energy and protein adequacy with greater energy and protein deficits. Enteral feeding should be withheld in patients with hemodynamic instability due to fear of bowel ischemia, which was related to derangement in intestinal microcirculation during shock (20). This concern was confirmed by Reigner et al. (33), where a higher risk of bowel ischemia and pseudo-obstruction was detected in shock patients receiving early EN in comparison to those prescribed early parenteral nutrition.

In brief, the review by Ramasamy et al. (34) reinforces several of the key strategies previously discussed to minimize FI. The author concluded that fasting in relation to surgeries should generally be limited to patients undergoing abdominal or thoracic procedures. Additionally, EN can be safely administered to patients in a prone position or those receiving neuromuscular blockade, and in a case where uncontrolled hemodynamics occur, initiation of EN should be withheld. It was also observed that the FI duration revealed a significant relationship with prolonged ICU stays, extended vasopressor use and longer mechanical ventilation duration, although the FI itself was not identified as an independent predictor for these outcomes. This is consistent with the findings by Peev et al. (15), who reported that those with FI had a 30% higher risk of prolonged ICU length of stay. These associations suggest that while FI may not directly determine clinical outcomes, it could still contribute to worsened outcomes, particularly in patients with high illness severity.

### 4.1 Differences in demographic, disease severity, and nutritional characteristics

Our study identified that those in the pre-protocol phase were older, had a larger risk of malnutrition, and were more ill upon ICU admission. They were also diagnosed with a greater number of concomitant co-morbidities, and the majority presented in a shock state, which could describe the higher disease severity score in the pre-protocol group. Our ICU is a mixed model that equally caters to medical and surgical admissions; however, the surge of COVID-19 cases in 2021 resulted in a significant increase in the number of available beds for critically ill COVID-19 patients, a reduction in the admission numbers for other medical and surgical patients, and the reallocation of these critically ill patients to other critical care areas. This shift in the patient’s profile contributed to the observed demographic differences. Obesity was detected in 70.7% of our COVID-19 patients. Zhang et al. (35) demonstrated that obesity was associated with an increased likelihood of presenting with severe COVID-19, developing ARDS, and admission into the intensive care unit, which could explain the significant proportion of COVID-19 patients with obesity and ARDS status upon ICU admission in the post-protocol group. Despite these baseline imbalances, subgroup analysis within the intergroup demonstrated that factors such as APACHE II score, admission type, and NUTRIC score were not associated with nutritional adequacy. To further assess the robustness of the results, multivariable linear regression analysis was conducted to evaluate the association between protocol implementation and nutritional outcomes. This approach was essential to reduce the bias in the interpretation of the protocol’s effect. After adjustment, protocol implementation remained significantly associated with earlier initiation of EN, an increase in nutritional adequacy, and reductions in both FI duration and energy deficit. Overall, these findings highlight that the observed improvements in nutritional characteristics could be attributable to the protocol, rather than due to differences in baseline characteristics, supporting the positive impact of structured and protocolized nutrition delivery, even in complex and evolving ICU populations.

Patients in our post-protocol group received enteral feeding significantly earlier, with more than 70% of the patients receiving feeding within 24 h after ICU admission, and this was associated with a greater energy goal than the pre-protocol group. The feeding efficiency in our study likewise showed significant improvement, reaching the targeted nutritional goal as early as day 2 after implementation of the protocol, with a greater proportion of patients achieving full feeding on day 4 of admission in the post-protocol group. These findings were consistent with a study by Kim et al. (17). In their study, the mean time for ICU admission to EN feeding was improved from 87.1 h to 35.8 h, *p*-value = 0.001, with a larger proportion of patients receiving EN within 24 h of ICU admission after implementation of the feeding protocol, 41% versus 59.6%, *p*-value = 0.002. The energy intake was also significantly improved from 38.3 to 52.2%, *p-*value = 0.037 (17). However, in contrast with Kim et al. (17), the time to accomplish 100% energy adequacy occurred later than day 2. One notable distinction of our protocol in comparison to theirs was that the feeding volume in our protocol was allowed to step up at least once every 8–12 h, as opposed to once a day. There was also an increased use of prokinetic prescription in addressing high GRV, which was evident in the post-protocol group, similar to Kim et al., where they used more motility agents after protocol implementation (34.3% vs. 53.7%, *p-*value = 0.001) (17). Our episode of feeding interruption related to high GRV and vomiting was documented at 13.7 and 16.6% before and after emphasizing the feeding protocol, in comparison with Lee et al., who documented a lower episode of GI intolerance at 7%. One of the reasons was due to a lack of understanding of the definition of GRV. We found that some of the healthcare personnel interrupted feeding even though the volume of GRV was less than 300 mL, which led to a 5.6% protocol violation rate in the post-protocol group. Yip et al. (18) concluded that the lack of understanding regarding management of gastric aspirates contributed to the 38% prevalence of FI due to GRV. These findings reflect the importance of active participation of ICU healthcare personnel and training in EN delivery, by emphasizing adherence to feeding protocol and identifying which patients that was suitable for early feeding.

Critically ill patients are mostly in a hypercatabolic state, especially in the acute phase of illness, for which the breakdown of muscle protein might exceed 1 g/kg/day (36). A protein deficit caused prolonged weaning, longer hospital stays, longer intubation period, and poorer general outcome (37). We showed a significant increase in the protein intake and adequacy after implementation of the feeding protocol, as seen in Doig et al. (38) and Heyland et al. (39), whereas Weijs et al. (40) and Compher et al. (41) showed that the odds of death and 28-day mortality decreased with greater protein intake. From the ESPEN guidelines, it was noted that the standard commercial product composition was not adequately enriched with proteins in comparison to the calorie content (42). Therefore, in our study, supplemental protein has been highlighted as part of the feeding strategy, and it was found that more than 50% of patients received extra protein supplement in the post-protocol period, which leads to an increase in the overall daily protein intake.

We identified that the prescription of energy-dense formula was commonly prescribed in the post-protocol phase to COVID-19 patients with ARDS. The use of an energy-dense formula did not contribute to better nutritional achievement or risk of overfeeding in our post-protocol patients. While full nutrition with energy-dense formula may be helpful for some patients, the decision on its usage as initial feeding should be performed with care and only in selected patients, such as those with acute respiratory failure, in order to avoid unintentional overnutrition (3, 43). It was shown that overfeeding was an independent risk factor for mortality in critically ill patients (40) rather than the prescription of an energy-dense formula alone. ESPEN guidelines in 2019 advised for a progressive increment of nutrition that does not exceed 70% of energy expenditure within 48 h to avoid overnutrition (44). In the same year, the TARGET trial demonstrated that the patients receiving energy-dense formula did not differ in the 90-day survival compared to those prescribed with routine formula (45).

Despite the implementation of a feeding protocol, the overall cumulative energy and protein deficits were comparable between groups. However, the sub-analysis of non-COVID-19 patients demonstrates improvement of nutritional deficits in the post-protocol group. Besides that, the interaction between protocol implementation and FI also showed more nutritional deficit in the post-protocol group, which suggests that although the protocol improved the energy delivery, patients remained vulnerable to the adverse impact of FI, particularly in the post-protocol phase. Importantly, subgroup analysis in non-COVID-19 patients also showed that every hour of FI in the post-protocol group led to an even greater increase in energy deficit and almost similar in protein deficit. This finding suggests that although the protocol improved overall nutritional delivery, patients remained vulnerable to the adverse impact of FI. Energy and protein deficits were also associated with delays in EN initiation and higher vasopressor requirements, reinforcing that the disease severity limits nutrient delivery. Therefore, these findings underscore the need for targeted strategies to reduce avoidable FI and support early EN. In addition to the previous discussions on strategies in reducing FI, Ramasamy et al. (34) summarizes other approaches, including the continuation of EN prior to extubation, evaluating vomiting and regular bowel movements as part of feeding tolerance instead of based only on GRV, starting EN within 24 h of abdominal surgery unless there is evidence of bowel obstruction, discontinuity, ischemia or ongoing peritonitis, and prescribing early nutrition via the gastric route rather than awaiting for small bowel access. Another measure to reduce nutritional deficit is by adopting volume-based rather than rate-based delivery of EN, as demonstrated by Bharal et al. (46).

### 4.2 EN protocol and outcome

We observed that the post-protocol group had longer EN days, ICU stay, and mechanical ventilation days. Martin et al. in the ACCEPT trial showed their evidence-based nutrition algorithms focusing on early provision of feeding similarly had an increased number of EN days. However, as opposed to our study, they observed that both hospital length of stay and mortality were reduced (47). It was shown that the volume-based feeding protocols are associated with a shorter ICU length of stay and reduced duration of mechanical ventilation than rate-based approaches (48). This is likely due to their capacity to compensate for FI by allowing catch-up volumes, thereby improving overall nutritional delivery. Unlike volume-based strategies, the rate-based EN approach used in our protocol may have limited the recovery of nutritional deficits after FI and could partially explain the longer ICU stays and prolonged mechanical ventilation even in non-COVID-19 cohorts. We also identified that delayed initiation of EN and prolonged vasopressor support were key factors associated with prolonged ICU stays and duration of mechanical ventilation. Evidence suggests that volume-based feeding supports more timely initiation of EN than the conventional rate-based approach, which may explain its effect on our ICU hospitalization and mechanical ventilation (48). Despite our analysis demonstrating the vasopressor duration was equivalent between groups, Herrera et al. supported that hemodynamically stable patients receiving early EN had shorter ICU stays and lower rates of ICU readmission (49). Furthermore, although COVID-19 infection did not affect the outcome of prolonged ICU and mechanical ventilation duration in our study, it is worth noting that severely ill COVID-19 patients were frequently associated with prolonged ventilation and feeding intolerance due to impaired gut function, and thus may also affect the overall length of mechanical ventilation and ICU stay, as demonstrated by Reignier et al. (33).

Despite the observed improvements in nutritional parameters after protocol utilization, such as energy and protein adequacy, earlier initiation of feeding in all cohorts and improvements of nutritional deficits in non-COVID-19 patients, these findings did not translate into corresponding improvements in hard clinical outcomes such as ICU length of stay and duration of mechanical ventilation. While optimal nutritional delivery is a fundamental component of supportive care in critically ill patients, unfortunately, it does not work in isolation. The complex interplay of disease severity, organ dysfunction, and timing of interventions may overshadow the benefits of improved nutritional intake. Our findings were consistent with Li et al., who demonstrate no significant difference in the duration of mechanical ventilation, although a modest reduction in ICU length of stay was observed, despite protocolized feeding strategies (10).

Our study also did not show that the feeding protocol has mortality benefits after implementation. Randomized controlled trials and systematic review suggested that the implementation of enteral nutrition feeding protocols was not associated with reduction in ICU mortality (10, 38, 50). The cause of ICU mortality was multifactorial, which warrant addressing of the different contribution factors. Our multivariate analysis revealed that underweight patients was associated with reduced survival, similar to study by Harris et al. The author analyzed 74,771 patients receiving early EN and demonstrated that the underweight patients had an 1.16 times increased risk of mortality compared to those with a BMI of 25 to 29.9 kg/m^2^ (51). We also detected that the longer duration of FI was correlated with longer duration of ICU stay, vasopressor requirement and mechanical ventilation. Peev et al. (15) found that the patients who experienced at least one interruption in their EN infusions were three times more likely to be underfed, accumulated a greater calorie deficit, prolonged hospital and ICU stay.

In our study, the illness-related FI and patients with severe COVID-19 were identified as mortality predictors, with a higher duration of FI in COVID-19 patients causing a larger energy deficit, longer ICU stay, duration in mechanical ventilation, and vasopressor use. It was found that patients infected with COVID-19 were at nutritional risk, and those with a higher nutritional risk had a 1.23 risk of higher mortality and longer hospital stay (52, 53). Chada et al. (54) also demonstrated that the energy deficit, disease severity, multiple co-morbidities, high nutritional risk, and prone ventilation were all correlated with COVID-19 mortality, while Drakos et al. (55) showed that feeding intolerance alone was an independent risk factor of ICU mortality. Based on this evidence, it was of paramount importance to establish EN as soon as possible, with extra efforts applied for the prevention of FI for COVID-19 patients.

Finally, to enhance the impact of the EN protocol, particularly in a setting of high care demands such as during the COVID-19 pandemic, several strategies should be considered. In summary, the protocol should be adaptable to the evolving clinical scenario, such as adapting low-dose trophic feeding during prone ventilation, which could reduce unnecessary FI. In addition to that, shifting from a rate-based to a volume-based approach may also improve nutritional deficits by allowing catch-up feeding. The procedural-related fasting also should be minimized and coordinated by implementing a fasting protocol, and finally, regular auditing of protocol adherence, along with regular staff training is essential to identify barriers and ensure consistent practice.

### 4.3 Limitations

Our study has several limitations. First, the single-center, observational study design and convenient sampling, with a significant proportion of COVID-19 pneumonia patients in the post-protocol group lead to an inevitable selection bias, as evident by differences in the demographic and disease severity state between the two groups. Thus, this limits the generalizability of results interpretation in the target population. Second, although FI was frequent in the pre-protocol group and should be avoided, we believed it was related to either stopping or cautious increment of enteral feeds in shock patients, which was appropriate clinical care in this setting. Furthermore, the contribution by illness related FI due to respiratory instability in COVID-19 patients also lead us to be unable to detect improvement in FI duration after feeding protocol implementation. Third, due to the observational nature of this study, our results cannot be interpreted as causative but rather as a well-controlled association. Finally, a better and more comprehensive protocol highlighting nutrition management of critically ill COVID-19 patients, such as nutritional therapy in a prone position, was required in order to improve feeding interruption in these populations.

## 5 Conclusion

The implementation of feeding protocol improved both feeding strategies and overall nutritional intake; however, it did not improve FI, except in non-COVID-19 patients. Illness related FI was associated with ICU mortality and duration of FI should be minimized, as it showed benefits toward improvement in nutritional deficits, and thus correlated with reduction of ICU stay as well as length of mechanical ventilation, including COVID-19 patients. Despite the limitations, the present study supported the implementation of an enteral feeding protocol in our center.

## Data Availability

The original contributions presented in the study are included in the article/supplementary material, further inquiries can be directed to the corresponding author.
